# Soil and Indoor Radionuclides Related to the Plymouth Pilgrim Nuclear Power Plant Area

**DOI:** 10.1177/15579018251371131

**Published:** 2025-11-01

**Authors:** Marco Kaltofen, Carolina L. Zilli Vieira, Eric Garshick, Petros Koutrakis

**Affiliations:** 1 Boston Chemical Data Corporation, Natick, Massachusetts, USA.; 2 Department of Environmental Health, Harvard T.H. Chan School of Public Health, Boston, Massachusetts, USA.; 3 Pulmonary, Allergy, Sleep and Critical Care Medicine Section, Veterans Affairs Boston Healthcare System, Boston, Massachusetts, USA.; 4 Harvard Medical School, Boston, Massachusetts, USA.

**Keywords:** indoor dust, nuclear power, radioactive particulates

## Abstract

Given the importance of internal dosimetry from inhaled or ingested radioactive particles attributable to nuclear fuels and fission, in this study, we analyzed samples collected from indoor home dust and surface soils in areas surrounding and up to 13 km from the Pilgrim Nuclear Power Plant (PNPP) in Plymouth, MA (USA). Indoor dust (*n* = 15) from homes (*n* = 10) and outdoor soil samples (*n* = 31, yielding a total of *n* = 46 original samples) were analyzed by γ-spectrometry, and α- and β-rate counting. Sixteen aliquots (16) from the 46 samples were analyzed by α-spectrometry for varying combinations of isotopic uranium and isotopic thorium, and by scanning electron microscopy with energy dispersive x-ray analysis. Detection of uranium and thorium confirmed that radioactive particles were of power plant origin. The presence of fission-related radionuclides was also confirmed by α- and γ-spectrometry. The highest activities of cesium-137 and lead-210 were observed in public areas outside of radiation protection zones near the PNPP, but evidence of fission-related radionuclides was found up to 12.1 km. Testing of soil and dust collected <1 km from the plant detected up to 20 times the background activity for cesium-137 and lead-210. Elevated thorium-234 and trace activities of cobalt-60 and uranium-235 were detected in surface soils sampled <1 km from the plant using γ-spectrometry, including uranium (>0.5%) and thorium (>5.0%) in microparticles. These isotopes were undetected or at background beyond these distances. This study suggests that radioisotopes released from nuclear power plants may be found in the soils and indoor dust of surrounding homes.

## Introduction

Previous studies have identified particle radioactivity (PR, radiation associated with airborne particulate matter) originating from operating nuclear facilities in dust samples from homes of workers or abutters of these nuclear facilities (Kaltofen and Bergendahl, 2010; Kaltofen and Gundersen, 2017; Kaltofen, 2019). For example, the advanced offgas (AOG) control system in a nuclear power plant is designed to capture radioactive gases vented during operation (GE, 2011). Leakage from the AOG system at Vermont Yankee produced contaminated surface soils with 36.3 to 151 Bq/kg of cesium-137 and 5.37 to 168 Bq/kg of cobalt-60 (VTDOH, 2010). Radioisotopes from these onsite surface soils can also be resuspended by weather or by disturbance by heavy equipment during decommissioning. Some fission products, such as compounds or hydrates of technetium or iodine, are semi-volatile and can also be released from heated wastewater or from the offgas system (Kim and Kruger, 2017).

There are over 100 nonoperating (retired) Nuclear Power Plants (NPPs) in the United States that are expected to be decommissioned in the coming years. Decommissioning (removal of primary sources of radioactivity) of the Pilgrim Nuclear Power Plant (PNPP), which operated between 1972 and 2019, located in Plymouth, MA, United States, started in 2019. This retired facility is in Plymouth County on the waterfront of the western end of Cape Cod Bay, approximately 60 km southeast of Boston, MA. The 517-acre plant is a General Electric boiling water reactor, with a capacity of 2028 MW thermal, 690 MW electrical (Pilgrim, 2012). Past studies have found that nuclear power plants release fission waste to the environment during routine operations. The Agency for Toxic Substances and Disease Registry (ATSDR) noted that in 1993, routine U.S. civilian boiling water reactors released 3.3 mCi of cesium-137 via air emissions and 580 mCi via water emissions. U.S. pressurized water reactors released 23 mCi via air and 2,850 mCi via water. On average, U.S. boiling water reactor power stations (including PNPP) released 29 mCi of cesium-137 to water, and 0.17 mCi to air in 1993.

This study hypothesizes that previous PNPP radionuclide releases could be assessed using samples of outdoor surface soils and indoor dust. Sampling and analysis sought evidence of nuclear fuel and fission-related isotopes (uranium-235, polonium-210, thorium, lead-210, and cesium-137) in samples of indoor dust and soil in the vicinity of the PNPP in Plymouth, Mass. The neutron activation product, cobalt-60, was also examined. Nuclear fuels contain a mix of uranium-234, −235, and −238, with a higher than natural ratio of uranium-235. Progeny isotopes such as polonium-210, lead-210, thorium-234, and others result from the radioactive decay of uranium (ATSDR, 2013). Polonium-210 is an α-emitter and an important driver of ionizing radiation risk (ATSDR, 1999). This isotope is a decay product of lead-210 (a β-emitter). Fission products, including cesium-134, cesium-137, and strontium-90, are produced by the nuclear reaction (ATSDR, 2004). Absorption of neutrons during nuclear fuel reactions produces cobalt-60 from stable cobalt-59 in the reactor (ATSDR, 2024). While uranium and its progeny also occur naturally, fission wastes, activation products, and uranium-235 in natural ratios are more typically industrial products (ATSDR, 2013).

Local geomorphology can introduce higher than typical activities of uranium, thorium, and their progeny. For this reason, the geospatial relationships among samples with activities greater than background, presumed sources such as NPPs, and local uranium or thorium mineralization require consideration.

The U. S. Agency for Toxic Substances and Disease Registry (ATSDR) notes that indoor dust is, in fact, an important component of soil and sediment ingestion (ATSDR, 2019). ATSDR notes that a release of chemicals into the environment can impact on all human exposure pathways. Chemicals in soil, dust, and sediment in residential settings are usually one of the more commonly evaluated pathways. Daily ingestion rates in ATSDR’s Public Health Assessment Guidance Manual (PHAGM) are 100 mg/day for adults, 200 mg/day for children, and 5,000 mg/day for children with soil-pica behavior.” The presence of radionuclides in soils and dust thus represents a source of potential internal radiation exposure (ATSDR, 2005).

To better understand the effects of both routine nuclear power plant operations and their decommissioning on the persistence of radionuclides in the environment that could impact human health, in this study, we measure nuclear fission-related radionuclides in home indoor dust and outdoor soils within 13 km of the PNPP. Environmental samples therefore reflect long term routine operation and decommissioning activities.

## Methods

### Indoor samples

Based on neighbor contacts provided by a concerned citizens group, we collected 15 dust samples from 10 homes within 13 km of PNPP. We also collected information regarding the age of the house and combustion sources (such as fireplaces or wood stoves). There were no reported smokers in the sampled homes. House age and other site data are in Table 1. Bulk indoor dust was collected from whole vacuum cleaner bags or by emptying the dust from canister (bagless) vacuums provided by the home resident. The 15 dust samples were stored in polyethylene Ziplock bags with identifying information attached. Indoor dust was mechanically removed from the vacuum bag of HVAC filters, according to the American Society for Testing and Materials (ASTM) methods D5438 (settled), D6966 (wipes), and D7144 (micro). Vacuum bag samples were extracted by removing all material collected between the coarse and HEPA bag filtration layers (Kaltofen, 2019). Nonbag samples of sufficient mass were collected by sieving canister contents using an ASTM #100 brass screen sieve (this passes only the particles with diameters smaller than 150 μm, (NIOSH, 2022)). Animal hairs, if present, were manually removed (Cizdziel and Hodge, 2000).

**Table 1.** Description of Dust and Soil Sites in Pilgrim Nuclear Power Plant Neighborhood

Home information	*N* (%)
*N*	
(*n* = 1, no data)	10
Age of home in years	
Median	55
Range	4–220
Heating source	
Radiator	2 (22.2)
Baseboard	5 (55.6)
Forced air	2 (22.2)
Open stove	5 (55.6)
Electric space heater	2 (22.2)
Gas space heater	2 (22.2)
Kerosene space heater	0
Wood stove	0
Fireplace	4 (44.4)
Type of fuel for heating	
Gas	6 (66.6)
Electric	0
Oil	3 (33.3)
Cooking fuel	
Gas	2 (22.2)
Electric	5 (55.6)
Other	2 (22.2)
Oven fuel	
Gas	8 (88.8)
Electric	1 (11.1)
Clothes dryer	7 (77.7)
Candles or incense	5 (55)
Pets (cats or dogs)	5 (55)

### Outdoor samples

Soil samples (31 of *n* = 46 total samples) were collected from outside each home (*n* = 10, 1 sample at each home) from low-lying or other locations where rainwater collected or flowed, in order to capture airborne particulate matter removed by precipitation. These were collected in Ziplock® polyethylene bags or LLDPE containers with identifying information attached. Soil samples were also collected from undeveloped locations (*n* = 21 of 31), including a coastal boreal forest located adjacent to the PNPP. The forested area slopes steeply toward the plant and the oceanfront. All soils were collected from the top 5 cm of the soil horizon using amber glass 250 mL precleaned jars that were supplied by the laboratory. Samples were collected without any trowel or device except the jar itself. A sufficient area was sampled to completely fill each container. Samples were sealed with a Teflon®/polyethylene lid. Background surface soil samples (*n* = 4) were collected from geographically similar areas in Massachusetts, more than 50 km from PNPP.

Outdoor soil and indoor dust samples were collected between April 8, 2024, and August 27, 2024. All samples met the exempt material activities as defined in 105 CMR Appendix A, Table A-2, and were handled and field-tested at remote or study site locations. These samples were analyzed by γ-spectrometry. The study was designated by the Institutional Review Board of the Harvard TH Chan School of Public Health as not requiring review as human subject research.

### Analytic methods

This study used multiple forms of radioactivity as markers of radioactivity attributable to PNPP. These markers included the fission waste isotopes cesium-134 and cesium-137. Fuel-related markers included isotopes of uranium, thorium, and radium, as well as the neutron activation product, cobalt-60, and the radioactive lead-210, a decay product of radon. Samples were also analyzed for radioactive (soil or dust) microparticles containing elements whose isotopes are radioactive, including uranium, thorium, radium, plutonium, or other transuranics. These elements are related to spent nuclear fuels. Although not necessarily specific to nuclear power plants, total α, β-, and γ-activities of indoor dust and outdoor soil were measured as markers of elevated activity.

Sample and data tracking were done using a Microsoft Excel spreadsheet. All samples were tracked using a unique identification number. Chain of custody documentation was maintained for all samples, including samples that were analyzed at commercial laboratories. Samples were prescreened to ensure compliance with 105 CMR 120.000 (Code of Massachusetts Regulations for the Control of Radiation).

All unique samples (*n* = 46 study and *n* = 4 background) were examined by γ-spectrometry, and activity levels were expressed on a becquerel per kilogram basis (Bq/kg). Twenty-one (21) samples were selected from the full set for additional total α and β^−^ analyses based on the presence of spectral peaks associated with nuclear-related isotopes (uranium, thorium, progeny, and cesium-137). Sixteen (16) of these 21 (21) was analyzed at the commercial laboratory for bulk radioisotope activity based on elevated α, β, or γ-activities, and these were standardized against a known activity of cesium-137. Two samples were also tested by SEM/EDS for radioactive microparticles (based on bulk radioisotope data).

All initial γ-spectrometry data was corrected for geometry (63 analyses of the original 46 samples) including duplicates and all aliquots of composited, whole and sieved air-dried samples). γ-photon analyses used Ortec® NaI (1.75” by 2.0” detector crystal model SP78W with 2K UCS-30 MCA and 1.5-inch thickness vertical lead shield stand, with 2-point energy calibration and resolution ±6 keV at 662 keV) photon detectors. NaI detection limits varied from 5 to 40 Bq/kg. Samples were transferred to tightly sealed 5 mL vials for analysis. Results are reported in Bq/kg using U.S. Environmental Protection Agency Method 901.1 Modified, Sodium Iodide (NaI) γ-spectrometry. The list of γ-detectable analytes relevant (but not necessarily actually detected) in this study included:

- Actinium-228
- Americium-241
- Bismuth-214
- Cobalt-60
- Cesium-134
- Cesium-137
- Potassium-40
- Lead-210
- Lead-212
- Lead-214
- Radium-226 and 228
- Thorium-234
- Thallium-208
- Uranium-235

An aliquot from each sample was analyzed for total α and total β activity using a Ludlum Model 3030 portable counter (background α = 4.6 counts per hour (cphr), 1σ detection limit = 6.7 cphr, background β = 2145 cphr, 1σ detection limit = 2206 cphr). Each sample counted weighed approximately 1 gram of dry weight and was counted with a 3-mm deep well-type planchette, yielding a 3-mm sample thickness. α-results were based on total area (4 cm^2^) as self-shielding prevents α counting by mass. β-results are in unit counts per gram of sample because self-shielding is minimal in 3 mm thick samples.

Sixteen (16) dust and soil samples with confirmed γ-detectable analytes underwent additional GeLi testing at Eberline Laboratories of Oak Ridge, TN (a commercial radiologically licensed radiochemical laboratory), using U.S. Environmental Protection Agency 908.0 Modified α-spectrometry for isotopic uranium (uranium-234, uranium-235, uranium-238) and isotopic thorium (thorium-228, thorium-230, thorium-232). These 16 samples were selected based on α- and β-counts more than 2σ above background counts or by the presence of γ-spectrometric peaks at 2X background or greater for γ-isotopes of interest (e.g., neglecting potassium-40, a common isotope found in natural minerals). Some samples, particularly the low mass dust samples, were not tested by all methods (γ-, α-, and/or β-), and untested isotopes are noted in Table 2. (Results are expressed in Bq/kg. GeLi γ-spectrometry detection limits were between 2.6 and 11 Bq/kg for most analytes. Lead-210 detection limits were from 37 to 74 Bq/kg.

**Table 2.** Bulk Radionuclide Results in Bq/kg with 1σ Counting Uncertainty (-- Not Tested)

Sample-Data in pCi/g	Matrix	Distance to NPP (km)	Cs-137	Pb-210	Ra-226	Ra-228	Th-234	U-235	Co-60
PLY1001D dust	Dust	5.15	ND <0.28	0.82 ± 0.48	ND <0.80	ND <1.01	5.21 ± 4.07	—	ND <0.41
PLY1003S soil 1	Soil	5.15	—	12.2 ± 2.7	—	—	—	—	—
PLY1004D dust	Dust	3.98	—	4.68 ± 0.69	—	—	—	—	—
PLY1012D FINES	Soil	3.98	0.15 ± 0.11	5.12 ± 0.69	0.33 ± 0.29	0.47 ± 0.45	ND <1.70	—	ND <0.18
PLY1013S FINES	Soil	3.98	ND <0.17	5.00 ± 2.53	ND <0.32	ND <0.57	3.30 ± 2.81	—	ND <0.23
PLY1016S FINES	Soil	1.93	ND <0.15	ND <2.22	0.71 ± 0.30	ND <0.47	ND <2.42	—	ND <0.15
PLY1017D FINES	Dust	0.82	0.37 ± 0.25	5.58 ± 1.23	—	—	—	—	—
PLY1019S FINES	Soil	0.83	ND <0.22	16.72 ± 4.94	1.15 ± 0.43	ND <0.80	8.07 ± 3.53	0.13 ± 0.10	ND <0.05
PLY1020S FINES	Soil	0.80	ND <0.12	5.86 ± 2.38	0.71 ± 0.27	1.13 ± 0.41	1.69 ± 1.37	—	ND <0.16
PLY-32–34-35 composite	Soil	0.69	3.09 ± 0.51	15.92 ± 4.98	0.80 ± 0.54	1.57 ± 1.29	ND <2.94	<0.05 ± 0.06	ND <0.27
PLY037S Fines. Soil	Soil	1.35	ND <0.16	ND <1.76	0.57 ± 0.28	ND <0.47	3.10 ± 2.14	—	ND <0.12
PLY038D Dust fines	Dust	1.48	ND <0.20	1.50 ± 0.53	ND <0.39	ND <0.83	3.95 ± 1.90	—	ND <0.22
PLY041S downspout	Soil	1.26	0.36 ± 0.18	10.51 ± 3.14	0.87 ± 0.30	1.28 ± 1.21	4.36 ± 1.73	<0.03 ± 0.06	ND <0.20
PLY045S Fines	Soil	12.15	0.38 ± 0.25	ND <2.81	1.11 ± 0.40	ND <0.76	6.41 ± 3.01	ND <0.05	ND <0.45
PLY055S-State Rd. Trails	Soil	0.16	1.01 ± 0.22	4.88 ± 2.25	0.54 ± 0.24	0.95 ± 0.67	ND <1.45	ND <0.09	0.17 ± 0.12
PLY-054–057 composite	Soil	0.16	1.08 ± 0.27	10.10 ± 2.57	0.55 ± 0.44	1.14 ± 0.80	3.51 ± 2.12	—	ND <0.12
Soil Background value	Soil	—	0.19	2.08	0.78	0.74	0.90	0.05	—

Cesium-137 in the background samples were below the detection limit (LLD), with the LLD given as background. All others are based on 2024 U.S. Environmental Protection Agency soil background values (US EPA, 2025).

The commercial laboratory analyzed lead-210 by the EPA-approved Eichrom method (PBW01, revision 2.1, 2014) using chemical resin-based separation followed by gas proportional counting. Polonium-210 is reported by assuming secular equilibrium with the parent lead-210 for this pilot study but can alternatively be explicitly reported via α-spectrometry.

Two dusts from these 16 samples tested for bulk radionuclides were also analyzed at MicroVision Laboratories of Chelmsford, MA (a commercial radiologically licensed ISO/IEC 17025:2017 accredited X-Ray laboratory) by scanning electron microscopy/energy dispersive X-ray analysis (SEM/EDS), using U.S. Environmental Protection Agency Method 600-R-02-070. These included one soil and one dust sample, which were selected based on γ-spectrometric results from the accredited laboratory using EPA-certified methods, and showing the presence of cesium-137, lead-210, or other isotopes of interest. This X-ray method determines the percent composition of elements with an atomic number of 5 or greater, including elements of interest such as cesium, strontium, sulfur, uranium, and thorium. Sample aliquots tested by this method had total weights of 2 to 4 mg each. Results are reported as photomicrographic images, X-ray spectra, and percent composition.

## Results

Soil and dust activities detected in this pilot study were highest at locations nearest the PNPP. Samples were collected up to 13 km distant, but the highest concentrations of cesium-137, thorium-234, cobalt-60, uranium-235, and radium-226 were all found within 1 km of the PNPP. Samples of soil particles collected near the PNPP perimeter contained radioactive microparticles with >0.5% of both uranium and thorium. Maximum total α- and total β-activities were found in a waterfront home indoor dust sample collected 5 km from the PNPP.

### Total α, β, and γ counting

Fifteen of 21 (15/21) house dust and outdoor soil samples were tested for total α-activity. Of these 21 samples, 15 had total α activities that were >2σ above the background samples’ α activity. Twelve of these 15 samples above background (12/15) were collected within 1.5 km of the PNPP.

Soil and dust samples collected within 1 km of the PNPP did have total α and β-counts at the +3σ level, but the highest α and the highest β-counts were found at a shorefront location 5.5 km from the PNPP. Samples from this location also had lead-210 activity at 451 ± 104 Bq/kg. This shorefront sample was the only sample collected outside of the 1.25 km radius from the PNPP that had more than two times the background activity for lead-210. Lead-210 is a β-emitter, and its short-term decay product polonium-210 is an α-emitter.

### Cesium-137 in dust and soil

Cesium-137 was considerably elevated compared to background in soils collected closest to the PPNP (3/14 samples). These surface soil samples contained between five and 20 times (37.4–114 Bq/kg) the background activity (7.0 Bq/kg) for cesium-137. Cesium-134 (half-life of 2.1 years compared to 30.2 years for cesium-137) was absent. The absence of detected cesium-134 in any soil sample in the study area is strong evidence that the presence of this fission product at elevated levels in Plymouth, MA, soils is due to historic PNPP releases rather than recent decommissioning activity (PNPP shut down on 5/31/2019). Figure 1 shows the unstandardized (estimated) cesium-137 by γ data (*N* = 63) compared with distances from the PNPP. Other bulk radionuclide results (including cesium-137) from the independent laboratory (*N* = 16) are given in Table 2.

**FIG. 1.**
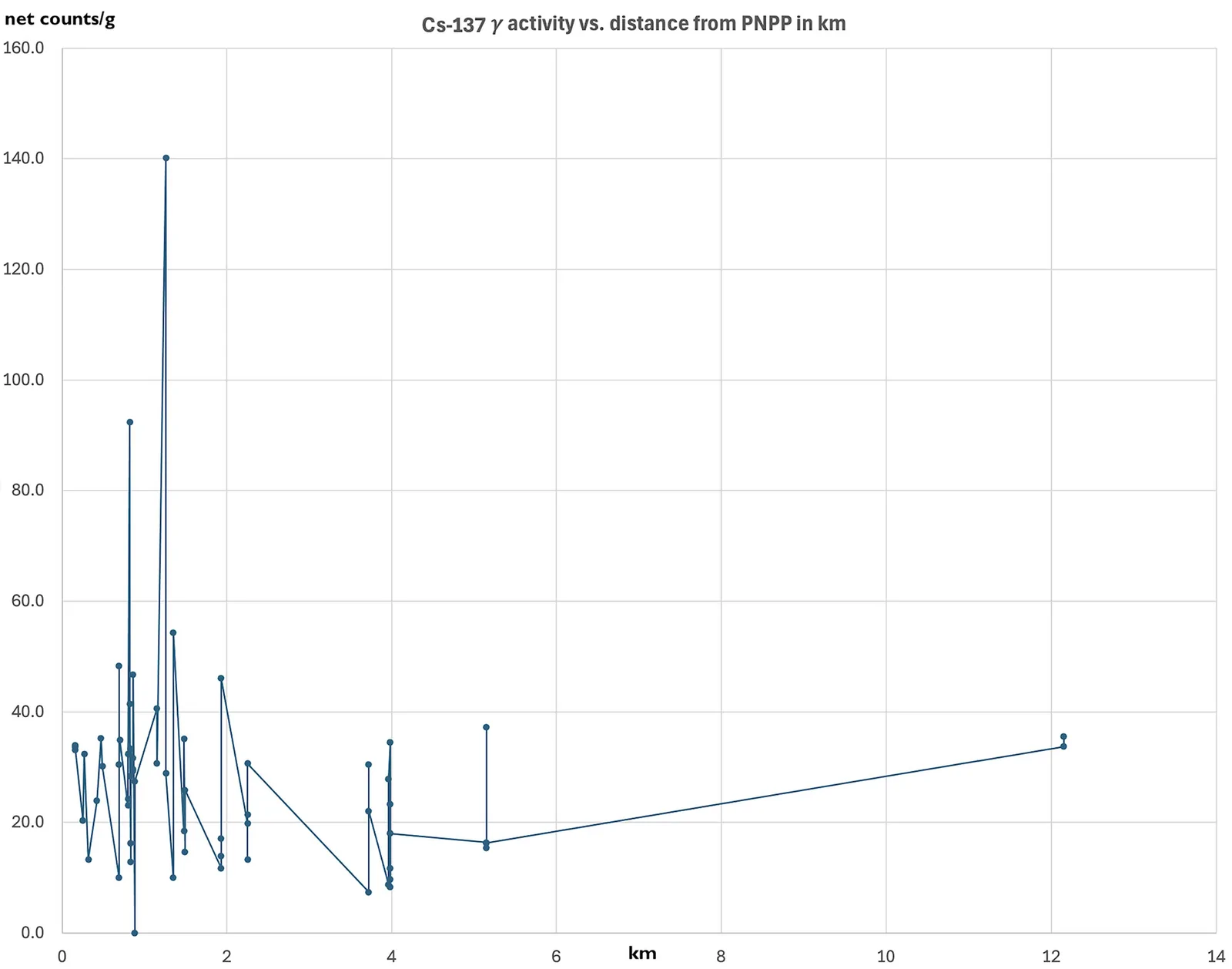
Unstandardized γ data for cesium-137 region of interest (*N* = 63) versus distance from PNPP in km. PNPP, Pilgrim Nuclear Power Plant.

The analyses of five coastal boreal forest soil samples lying between 0.16 and 0.83 km from the plant found cesium-137 activities in soils ranging from 40 ± 10 to 114 ± 19 Bq/kg dry weight. These activities greatly exceed the expected soil background. The bulk of the USA background cesium-137 is the result of atomic test detonation fallout from the 1960s. Background soil results for this study were undetectable, with presumed cesium-134 and cesium-137 activities less than the detection limits of 5.5 to 9.3 Bq/kg.

### Lead-210 in indoor dusts and outdoor soils

Testing in public areas near the PNPP (*n* = 16 samples) also found significant amounts of lead-210 (up to 619 ± 183 Bq/kg vs. the published U.S. soil background of 77 Bq/kg, which is based on analyses undertaken by the U.S. EPA Superfund Program (US EPA, 2025). Lead-210 is a decay product of uranium-238, thorium-234, and radium-226 which are all found in spent nuclear fuels. Of all homes tested, this maximum lead-210 result came from the home that was closest to the PNPP. This same home was also the only one where uranium-235 (a fissile nuclear fuel component) was found at approximately two times the expected background (US EPA, 2025) (4.8 ± 3.7 Bq/kg vs. background of <1.8 Bq/kg). All other samples had undetectable uranium-235 results.

Testing in soil in public areas near the PNPP also found significant amounts of lead-210. These isotopes are all found in burned nuclear fuels. Lead-210 activity in study samples was strongly related to distance from the PNPP. Six of the eight highest lead-210 results were found within 1 km of the PNPP. The maximum result (619 ± 183 Bq/kg) was found in a surface soil sample 800 meters from the PNPP and in a residential location. The next highest surface soil result (589 ± 184) Bq/kg) was found in a forested area 630 m from the PNPP.

Of the 10 home site soils tested in this pilot study by γ-spectrometry, the maximum thorium-234 (299 ± 131 Bq/kg), uranium-235 (4.8 ± 3.7 Bq/kg), and radium-226 (42.6 ± 3.9 Bq/kg) values were all at the same location (0.83 km from PNPP). The uranium-235 result at this location is approximately two times the mean U.S. soil background (<1.8 Bq/kg) and was undetected in all other study samples (US EPA, 2025). Conversely, soil and indoor dust from a control site located 12.1 km from the PNPP had undetectable lead-210 activities, both less than 104 Bq/kg. Likewise, 43 of the 52 dust and soil samples (46 original samples plus 6 sieved aliquots) had lead-210 activities below 185 Bq/kg (Fig. 2).

**FIG. 2.**
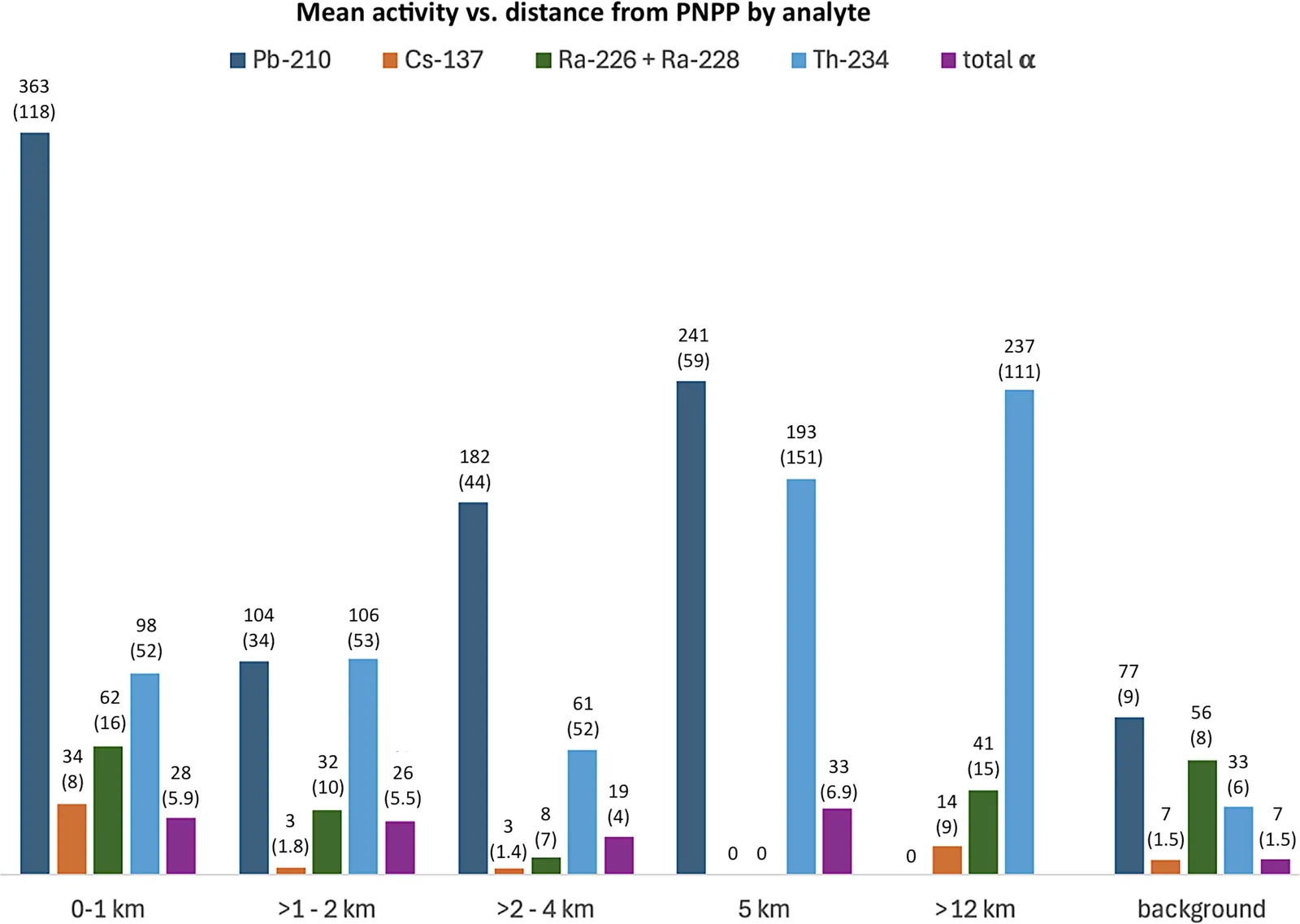
Histogram shows distance (km) from PNPP versus mean sample activity (Bq/kg) for isotopic data and total α.

Cobalt-60, a neutron activation product common to nuclear reactors, was found in 1 of 13 samples at the trace level of 6.3 ± 4.4 Bq/kg. The positive sample was the soil sample collected nearest the PNPP.

Two samples (one soil and one indoor dust sample) were collected near the perimeter of the PNPP. These samples were found to have the greatest activities above background for cesium-137 and lead-210 as analyzed by the independent laboratory. The outdoor soil sample was collected on a public recreation trail across the street from the PNPP (160 meters from the plant), and the dust sample came from inside a home in the closest residential area to the plant (820 meters from the plant). These samples were analyzed by the independent X-Ray laboratory using SEM/EDS to determine the elemental composition of particulate matter containing uranium, thorium, radium, and other high atomic number elements.

This soil sample contained 115 Bq/kg of cesium-137, as well as particles containing up to 0.54 ± 0.04% strontium. The nearest house sample (0.83 km from the PNPP) had similar levels of strontium in dust particles. Strontium was not detected in background samples, except for naturally occurring sulfur-strontium compounds, nor was cesium-137 detected in background samples. The background value given for cesium-137 in Table 2 is the detection limit. This was borne out in aliquots from a soil sample analyzed both by the X-ray analysis and by high-resolution γ-analysis.

Based on the X-ray data for the subset of soils collected near the perimeter of the PNPP, there are multiple radioactive microparticles per mg of soil. The particles were about 10 to 20 μm in size and contained percent concentrations of uranium and thorium, while the bulk samples from which these radioactive microparticles came contained 37 to 111 Bq/kg of cesium-137. Strontium (0.5 to 1.3% by weight) was found sparingly in particulate matter for both the nearest soil and nearest house dust samples. Strontium sulfate (SrSO_4_) is by far the most common strontium-containing mineral (USGS, 2025). However, sulfur was absent in the soil sample containing strontium.

Figure 3 presents an X-ray photomicrograph showing an amorphous mass adhering to a soil particle. In backscatter mode, SEM/EDS images are bright white for thorium and other high atomic mass elements. The white areas in Figure 4 are from a backscatter image showing that the adhering material in Figure 3 is brightly lit, evidence of high thorium and uranium content throughout this adhering material. The underlying material, however, is dark, meaning that neither thorium nor uranium is present in the surrounding soil particles. These two images are consistent with soil contamination from a microscopic but highly radioactive contaminant particle. The SEM/EDS spectrum for this mass (Fig. 5) found that it contained 5.18 ± 0.17% thorium and 0.97 ± 0.03% uranium. The particles detected in soils adjacent to the PNPP had 1% to 7% thorium (mean = 5%) and 0.5% to 1% uranium.

**FIG. 3.**
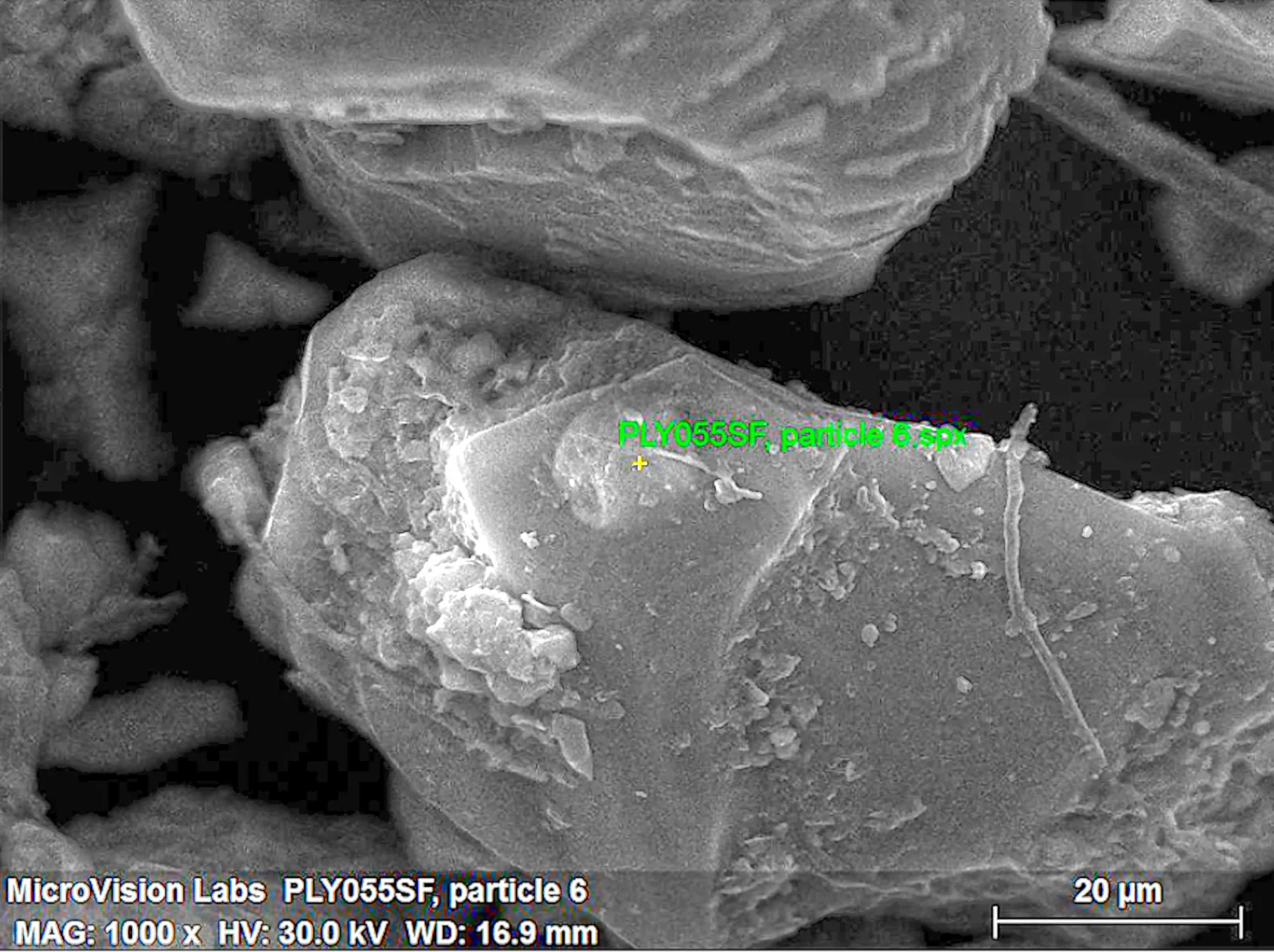
Secondary electron photomicrograph of particle with 5.2% thorium and 1.0% uranium collected adjacent to PNPP.

**FIG. 4.**
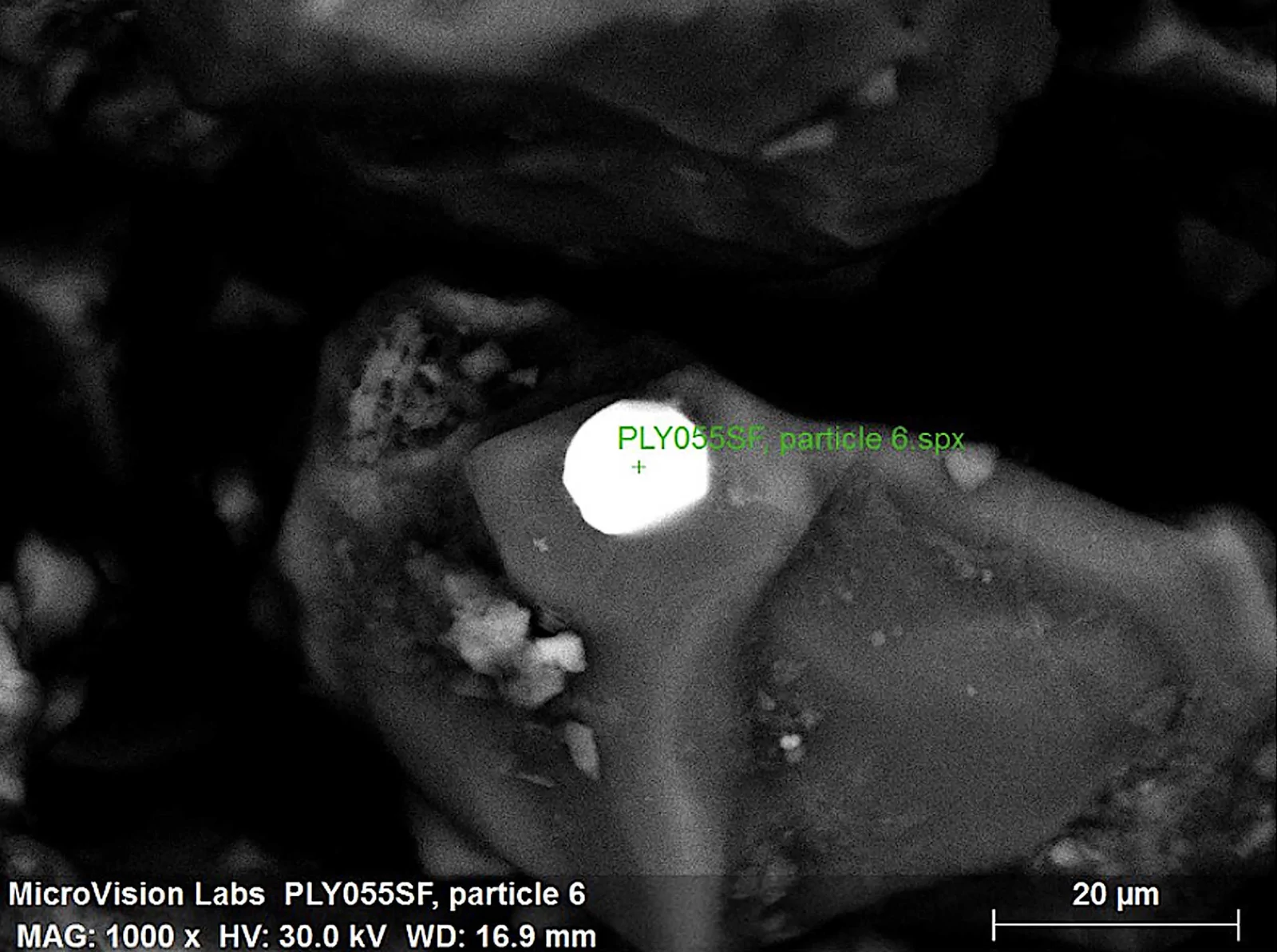
Backscatter electron photomicrograph of particle (central bright spot) with high thorium deposit on soil matrix.

**FIG. 5.**
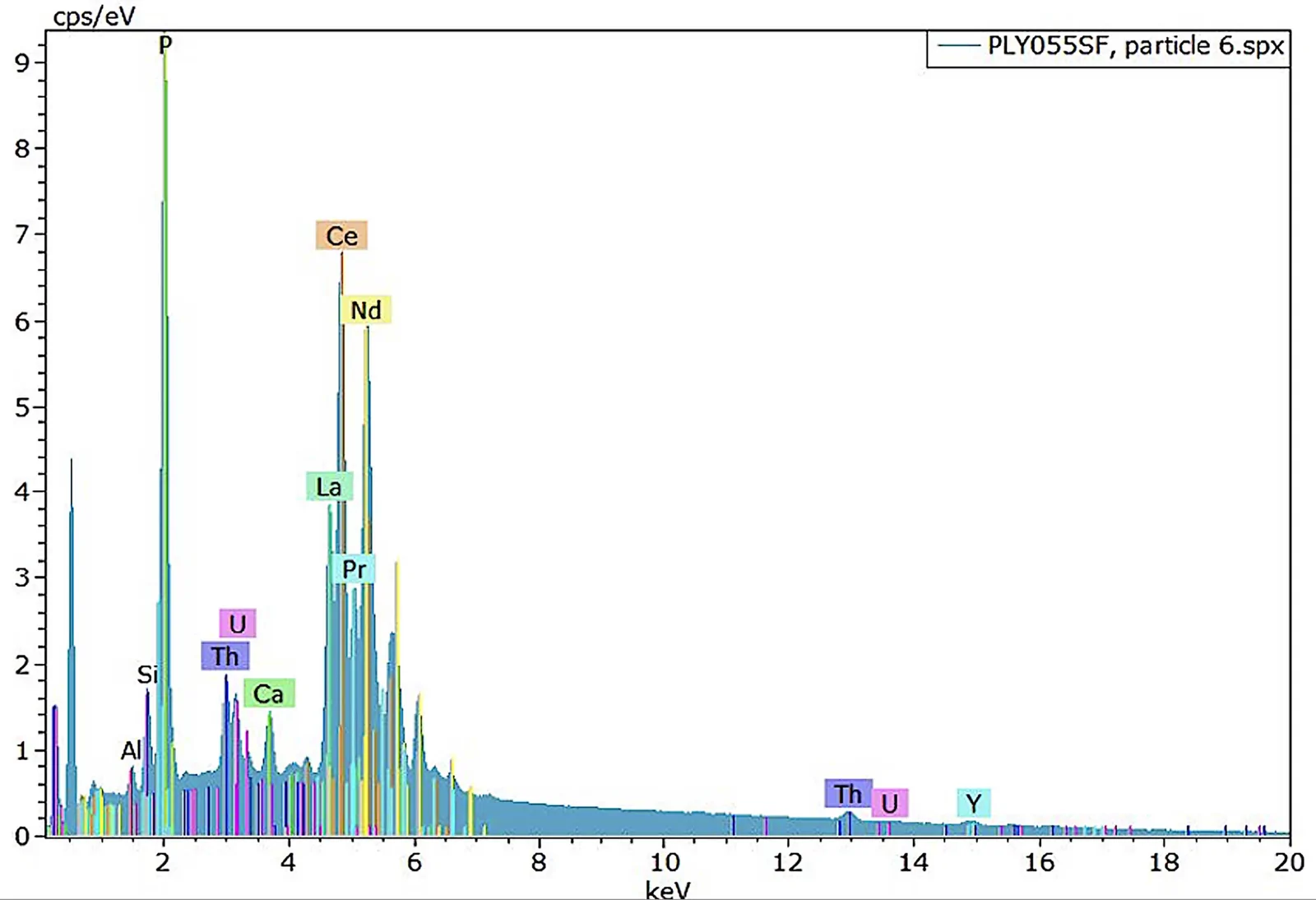
Scanning electron microscopy/energy dispersive X-ray spectrum for high thorium/uranium particles.

## Discussion

The test results show strong evidence of nuclear fuel and fission-related isotopes (uranium-235, polonium-210, thorium, lead-210, and cesium-137) and limited evidence of neutron activation products (cobalt-60) in samples of indoor dust and soil in the vicinity of the PNPP in Plymouth, Mass. The presence of cesium-137, a nuclear fission product unrelated to naturally occurring radioactive material, suggests that this set of detected isotopes is likely to result from the PNPP’s operations or decommissioning.

In 2016, researchers reported surface soil cesium-137 activities ranging from 12.2 to 151 Bq/kg (median = 36 Bq/kg, *n* = 10) within the perimeter of the Vermont Yankee NPS (VTDOH, 2010). Cesium-137 is a common nuclear fission product. The MA DPH reported similar results on the PNPP site itself, with cobalt-60 (maximum 42.6 Bq/kg at a depth of 6.5 feet vs. Nondetect in typical background soil) and cesium-137 (maximum 92.1 Bq/kg at a depth of 6.5 feet vs. 0.37 to 37 Bq/kg in soils nationwide due to historical fallout from atomic bomb testing) were present in subsurface soil samples collected at 3, 5, and 6.5 feet below ground surface (MADPH, 2013). Researchers at the Vermont Yankee NPP also found in surface soil cesium-137 at similar activities. This suggests that this elevated cesium-137 finding is not unique to PNPP.

Lead-210 is a decay product of radium-226 via radon-222, a gas. This means that lead-210 can be found in unsupported form in secular disequilibrium from its parent radium-226. This unsupported lead-210 result was seen in all the soil and dust samples with elevated lead-210 activities. None of the soil and dust samples in the study set had radium-226 activities >42.6 Bq/kg, while lead-210 activities exceeded 555 Bq/kg at multiple locations. In addition, U.S. Environmental Protection Agency figures (USEPA, 2014) show that background lead-210 activity in soils is closer to 77 Bq/kg. This is strong evidence that the lead-210 found clustering near the PNPP is the result of the decay of airborne radon-222, potentially from the reactor offgas system or from spent fuel gaseous releases. The presence of lead-210 is of special public health concern because it produces a short-term decay product, polonium-210, an α-emitter that is typically in secular equilibrium with its lead-210 parent.

Indoor smoking and fossil or wood fuel burning can contribute to indoor dust lead-210 activities. None of the residents who responded to questionnaires reported being smokers. In addition, the radionuclides identified are not associated with fossil fuel combustion.

Radioactive microparticles were detected in soil samples collected within 1.0 km of the PNPP. These were detected at a rate of approximately one to two radioactive particles per milligram of fine (passing a 150 μm screen) particulate matter. Elemental analyses by SEM/EDS show that these were particles composed of thorium with uranium and rare earth elements. Some thoriated particles also contained strontium. Since all isotopes of thorium and uranium are radioactive, all these microparticles containing thorium and uranium are radioactive.

The PNPP perimeter soil sample has many radioactive microparticles containing thorium or both thorium and uranium. In addition, they contained rare earth lanthanides [Ce (cerium), La (lanthanum), Sm (samarium), Nd (neodymium)] that generally follow the composition of thorium monazite minerals. Lanthanides are also a common constituent of fission waste. The presence of these elements in our samples suggests an industrial rather than natural origin for the radioactive microparticles detected. Nuclear fuel reactions tend to produce equal amounts of radioactive cesium and radioactive strontium (Kavasi et al., 2023). It is expected to find some amount of strontium-90 and its short-term decay product yttrium-90 in soil and indoor dust particles that contain cesium-137.

Soil particles with thorium and uranium, once inhaled, would yield an internal tissue dose on the order of 50 ± 12 rads per year. This dose is reduced by 50% as this geometry results in half of the α emissions becoming absorbed in the underlying mineral particle (meaning that the linear range of the emitted α particles is much smaller than the thickness of the material obstructing the bottom face of the thoriated particle, creating a geometry where 50% of emissions are absorbed). Thorium monazite minerals normally exhibit monoclinic crystallization (Clavier et al., 2011). This amorphous appearance suggests that the thorium and uranium detected are industrial rather than natural in origin. While thorium monazite is not rare, thorium-uranium monazite is substantially less common. Typical thorium monazite has 0.5% to 5%, thorium and uranium content is rarely above 0.5% (USGS, 2009; USGS, 1967). The presence of isolated particles with high percentage concentrations of uranium and thorium in an area without related mineralization suggests that the NPP is a likely source of these radioactive microparticles.

Maps of mineralization in Massachusetts show local deposits of thorium monazites in Weymouth, Hingham (both are coastal locations north of Plymouth, MA), and Ayer. Importantly, however, this thorium-containing mineral has not been previously found in Plymouth, which is a well-studied area mineralogically (Gleba, 2024). This is evidence that the thorium and uranium-containing particulate matter is related to releases from the PNPP.

Airborne and resuspended radioactive microparticles from soils and dusts are readily transported by winds and can transport particulate materials from radiation protection zones to outside areas (Friedlander, 2000). The relative locations of the PNPP, points of maximum lead-210 and cesium-137 activity, confirmed percent-uranium and percent thorium in microparticle locations, and maximum average wind speed vector are shown in Figure 6.

**FIG. 6.**
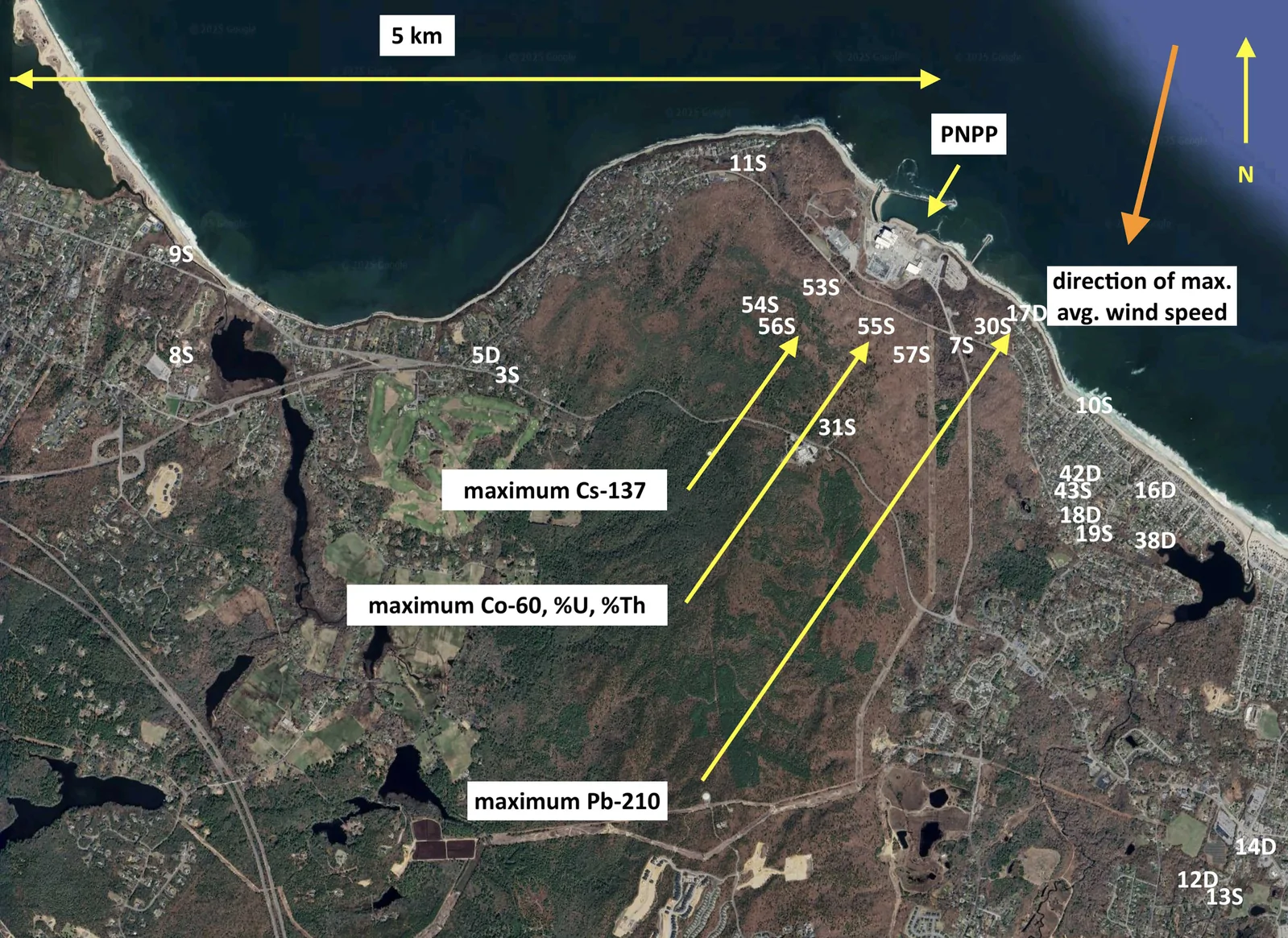
Study samples, maximum impact locations, and location of PNPP.

This study was designed to identify nuclear power station-related radioisotopes in soils and dust in the vicinity of the permanently offline Plymouth Pilgrim Nuclear Power Station. The work included six different analytical methods used on both soil and dust samples and collected over a wide geographic area. The 46 unique samples collected were sufficient to identify isotopes of concern and to show that these isotopes were found at significantly elevated activities in areas closest to the PNPP.

However, the number of samples is limited compared to the multiple variables in the collection and analytical scheme. Future studies would benefit from a larger number of samples as well as consistent use of α-spectrometry, particularly for polonium-210, an isotope of significant concern. Examining the spatial patterns of PNPP-related radioactive materials will require additional samples that focus on the primary isotopes of concern (cesium-137 and lead-210) identified in this study. Additional SEM/EDX samples will also be required to assist in quantifying the dose to people in the impacted area. Given the number of nuclear facilities with similar features and histories as the PNPP, this same type of single-site soil and dust investigation is also appropriate for NPPs outside of this study area.

## Conclusions

This study found strong evidence of nuclear fuel (uranium, thorium, and lead-210) and nuclear fission-related (cesium-137) isotopes in samples of indoor dust and soil in the vicinity of the PNPP in Plymouth, Mass. There is also evidence for uranium-235, cobalt-60, and polonium-210 (based on equilibrium considerations) in particulate matter.

The occurrence and concentration of radioactive cesium-137 and lead-210 were higher near the PNPP. The radioisotope activities were consistent with known radioactive contamination on the PNPP site (within the radiation protection zone). However, all these results in this study were found in public or residential areas outside of radiation protection zones. Inhalable or ingestible radioactive microparticles containing thorium and uranium were found in particulate matter near the PNPP.

Based on the X-ray data for soils near the perimeter of the PNPP, there are multiple radioactive microparticles in this soil sample per mg of soil. The detected particles were each about 10–20 μm in size and contained percent levels of uranium and thorium, and 37–111 Bq/kg of cesium-137. Despite the small number of samples collected, the presence of these radioactive materials associated with the PNPP in particulate matter outside of this nuclear power station in Plymouth, MA, represents a significant potential source of internal radiation dose to people in the surrounding area.

## Data Sharing

Data are available for sharing upon reasonable request.

## Authors’ Contributions

M.K.: Responsible for conceptualization, data curation, methodology, investigation, writing (original draft, editing). C.L.Z.V.: Responsible for investigation, supervision, funding acquisition, writing (original draft, review and editing). E.G.: Responsible for writing (review and editing), validation of analytical results, and formal analysis of study data. P.K.: Lead scientist responsible for conceptualization, supervision, writing (review and editing), funding acquisition, resources, and project administration.
